# Socioeconomic gradient in mortality of working age and older adults with multiple long-term conditions in England and Ontario, Canada.

**DOI:** 10.23889/ijpds.v7i3.1898

**Published:** 2022-08-25

**Authors:** Anne Alarilla, Mai Stafford, Luke Mondor, Jay Hughes, Hannah Knight, Anna Pefoyo Kone, Walter Woodchis

**Affiliations:** 1 The Health Foundation; 2 Institute for Clinical Evaluative Sciences; 3 Health System Performance Network; 4 Department of Health Sciences, Lakehead University; 5 Institute of Health Policy Management & Evaluation, University of Toronto

## Objectives

There is mixed evidence on the influence of number of conditions on inequalities in mortality, we explored the association between number of conditions and deprivation on mortality whilst assessing the difference between working age and older adults. We replicated the analysis in England and Ontario, to provide a cross-jurisdiction comparison.

## Approach

We used individual level-linked data from primary care activity, secondary care and mortality data in England and Ontario. For both jurisdictions, we took a random sample of 600,000 adults from 1 January 2015 and followed them up till 31 December 2019. We used cox proportional hazard to test the influence of deprivation (measured using area-level deprivation in deciles) and number of conditions (measured at baseline and capped at six conditions) on survival. Age and sex were also measured at baseline. Analyses were stratified by working age (18-64 years) and older adults (65+ years) and were repeated for England and Ontario.

## Results

Deprivation gradient in mortality rate was steeper in working age than older adults in both jurisdictions. Number of conditions was associated with increased mortality rate, this was stronger for working age than older adults, in England (working age HR = 1.60, 95% CI 1.56,1.64 and older adults HR= 1.26, 95% CI 1.25,1.27) and Canada (working age HR=1.69, 95% CI 1.66,1.72 and older adults HR= 1.39, 95% CI 1.38,1.40). After accounting for number of conditions, the mortality rate associated with deprivation decreased but remained significant. The interaction between number of conditions and deprivation showed that adults with more conditions have a higher mortality rate and those living in deprived areas also have a higher mortality rate but having more conditions attenuates the deprivation gradient in mortality.

## Conclusion

Number of conditions contribute to higher mortality rate and inequalities in morality, this is stronger for working age than older adults in England and Ontario. The fragmented health-care system may be contributing to poorer outcomes, further research should help identify which part of the pathway is driving these inequalities further.

